# Effect of on-farm hatching and elevated platforms on behavior and performance in fast-growing broiler chickens

**DOI:** 10.1016/j.psj.2025.104910

**Published:** 2025-02-17

**Authors:** Julia Malchow, Roos Molenaar, Mona F. Giersberg, Ingrid C. de Jong, Bas Kemp, E. Tobias Krause, Lars Schrader

**Affiliations:** a Institute of Animal Welfare and Animal Husbandry, Friedrich-Loeffler-Institut, Celle, Germany; b Adaption Physiology Group, Wageningen University and Research, Wageningen, The Netherlands; c Department Population Health Sciences, Faculty of Veterinary Medicine, Utrecht, University Utrecht, The Netherlands; d Department Animal Welfare and Health, Wageningen Livestock Research, Wageningen, The Netherlands

**Keywords:** activity, body temperature, chick quality, enrichment, walking ability

## Abstract

Alternative hatching systems compared to conventional hatchery-hatched systems showed positive effects on welfare of broiler chickens. In order to investigate an additional positive effect of elevated platforms, two hatching methods (on-farm [OH] vs. hatchery-hatched [HH]) and two environments from the first day onwards (with elevated platforms [enriched] vs. without elevated platforms [control]) were combined and investigated using a 2 × 2 factorial design. In three consecutive trials, the combination of the four treatments were repeated eight times each. One thousand six hundred fast-growing broiler chickens (Ross strain) were reared in a mixed-sex system. Chick quality was assessed at hatch and performance parameters and behavior parameters were measured during the entire rearing period of 35 d. For the statistical analysis, LME's and GLMM's were used depending on the data. In general, hatching system and housing environment showed no interaction. There were no differences in hatchability between treatment groups (p=0.93). However, OH chickens showed a higher body weight throughout the rearing period (all p<0.001). OH chickens had a lower body temperature than HH chickens (p=0.002) during the rearing period. OH chickens compared to HH chickens tended to show a higher usage of elevated platform at night (p=0.07). The enriched groups showed higher activity (p<0.0001), but no improved walking ability (p=0.82) than the control groups. The differences in performance and behavior were low between hatching systems and may be related to the short period of feed and water deprivation and the lack of long commercial processing and transportation procedures in the HH treatment group in our experiment. Overall, both on-farm hatching and elevated platforms can lead to an improvement of performance and activity parameters and, thus, an improvement of certain aspects of animal welfare but both factors do not seem to interact with each other.

## INTRODUCTION

The total incubation period of chicken eggs is around 21 days (510 hours), whereby the first chicks already start to hatch from embryonic day 19 (=ED 19) onward (461 hours onwards; Tong et al., 2011). This so-called hatch window, i.e. the time between hatching of the first and the last chick within a batch of eggs, is between 24 to 48 hours in commercial hatcheries (Tong et al., 2013). During this hatching process, chicks usually have no access to feed and water and are kept often in complete darkness. Routine processing procedures at the hatchery, such as loading and transporting, can further increase this period of feed and water deprivation, and expose chicks to noise and handling that can cause short- and long-term stress responses (Hedlund et al., 2019). Moreover, feed and water deprivation that exceeds 48 hours can negatively affect post-hatch chick growth (de Jong et al., 2017), development of the gastrointestinal tract (Lamot et al., 2014; Dieryck et al., 2022) and development of the immune system (Panda et al., 2015).

To avoid handling and various stress factors in a hatchery and to provide early feed and water, 18-day incubated eggs can be transported and hatched on the farm. In this alternative hatching system, chicks have immediate access to feed and water after hatching. Several commercial on-farm hatching systems are currently available, whereby the technical design and the degree of automation differ. In some systems, eggs are placed on the floor either by a machine in piled-up nests consisting of the bedding material in the barn (e.g. NestBorn®, Hoegaarden, Belgium) or they are placed in hatching trays on the bedded floor (e.g. One2Born®, Uden, The Netherlands). In other systems, egg trays are placed in an elevated rail system above the litter (e.g. X-Treck®, Vencomatic Group, Eersel, The Netherlands). In the latter systems, the chicks can reach an intermediate level after hatching from which they can move to the floor where feed and water are provided.

On-farm hatching has been found to positively affect performance parameters expressed by lower mortality (de Jong et al., 2020), higher live body weight (Souza da Silva et al., 2021), and improved animal welfare and health as indicated by an improved foot pad quality (de Jong et al., 2019) compared to chickens hatched in commercial hatcheries. On-farm hatching can also affect the behavior of the animals. In general, the activity seems to be lower in these animals (Giersberg et al., 2023; Jessen et al., 2021a) as a result from on-farm hatching. However, other behavioral traits are also affected but seem to be influenced from the respective breed used for on-farm hatching. Fast growing broiler chickens have a higher fearfulness at on-farm hatching (Giersberg et al., 2020) and in slower growing broiler chickens’ fearfulness is lower (Jessen et al., 2021a) when hatched on-farm compared to conventional hatched broiler chickens.

In general, fast-growing broiler chickens are often reared in rather barren environments only containing water and feeder lines, and litter material provided in floor housing systems. A more complex environment including enrichment, e.g. elevated structures, may positively affect affective states in broiler chickens by reducing their fearfulness (Tahamtani et al., 2018). A lower level of fearfulness can decrease stress and mortality, and can lead to reduced sporadically occurring arrhythmia (Rupert and Zipes, 2005) and startle responses, indicating that the animals may have improved abilities to cope with new stimuli in their environment potentially contributing to an improved welfare (Anderson et al., 2021).

Other behaviors can also be positively influenced by structural elements in the barn such as elevated structures (reviewed by Riber et al., 2018). Elevated structures can support the roosting behavior of layer chickens as well as broiler chickens (Malchow et al., 2019a), which likewise enhances highly motivated exploration behavior of young chickens. In addition, elevated structures can help birds to avoid or to escape from agonistic interactions with conspecifics (Cordiner and Savory, 2001). Providing elevated platforms can lead to positive physical effects, like increased activity (Bailie et al., 2013; Güz et al., 2021) and may reduce leg problems in broiler chickens (reviewed by Pedersen and Forkman, 2019) by improving bone strength and increased muscle use (Yan et al., 2014; Kaukonen et al., 2017). Usage of elevated platforms can result in drier feet of chickens due to reduced exposure times to the potentially wet bedding material, leading to lower occurrence of foot pad dermatitis (de Jong and van Harn, 2012; Hongchao et al., 2014). Less contact with bedding material may also result in reduced dirtiness of the broilers’ abdominal plumage (Zhao et al., 2012). Effects of elevated structures on the live weight of broiler chickens throughout the rearing phase are not consistent between different studies and range from no effects of elevated structures on live weights (Malchow et al., 2019b, Jones et al., 2020), to decreasing (Güz et al., 2021) and increasing weights (van der Eijk et al., 2022). Mortality does not seem to be affected by elevated structures (Bailie and O'Connell, 2014, Güz et al., 2021).

So far, there are no studies investigating health and behavior of broiler chickens under conditions combining different hatching systems with elevated platforms. In this study, two different hatching systems (hatchery-hatched [HH] *vs.* on-farm [OH]) and two housing systems (enriched *vs*. control) were therefore compared in a 2 × 2 factorial design. It is hypothesized that both on-farm hatching and elevated platforms have a positive effect on broiler behavior and performance in fast-growing broiler chickens, thereby improving animal welfare and health, and especially the interaction between both have a beneficial effect.

## MATERIAL AND METHODS

### Ethical Statement

The experiments were carried out with the approval of the Lower Saxony State Office for Consumer Protection and Food Safety (LAVES, Oldenburg, Germany, file number: 33.8-42502-04-20/3473). The investigation was performed in compliance with national (TierSchNutztV, 2021) as well as European regulations (RL 2007/43/EG) at the research station of the Institute of Animal Welfare and Animal Husbandry, Friedrich-Loeffler Institut, Celle, Germany.

### Animals and Housing

The experiment was performed using a 2 (hatching system) x 2 (environment) factorial design. In three consecutive trials, the four treatments from the 2 × 2 design were repeated eight times each. Fast-growing broiler chickens (Ross 308, Aviagen®) were hatched in one out of two hatching systems: 1) conventional hatcher, i.e. without light, water and feed until arrival at the experimental barn (hatchery-hatched, HH) with occurred at expected ED 21; 2). On ED 18, fertile eggs were transferred to the experimental barn (same for the rearing period post-hatch) with 24 h light on. The animals had the opportunity to access feed and water immediately after hatching (on-farm hatched, OH). During the entire rearing period from day 0 to 35, half of the animals were kept in a conventional housing environment (control) and the other half of the animals were reared in an environment with elevated platforms (enriched).

*Hatching procedure.* All first-grade hatching eggs were obtained from a commercial hatchery (storage period < 1 week) and from a young parent stock with ages ranging from 27 to 32 weeks. At the research facility, 12 trays for trial 1 (T1), 12 trays for trial 2 (T2), and 8 trays for trial 3 (T3) were filled each with 62 to 63 eggs (expected hatchability of 80%) in order to be able to rear 50 hatched chickens (25 females and 25 males) per pen. Before the incubation process began, the eggs were already placed in groups on separate trays, considering the treatment groups. Thereafter, there was no exchange or other allocation of the eggs and later groups.

At the facility, the incubation process took place in a small-scale setter and hatcher (Petersime Vison Typ 96, Petersime, Zulte, Olsene, Belgium) with automatic computer control. For the first 18 ED, all eggs were placed per pen in a setter trays and randomly allocated in the setter. The climate was controlled by applying a standard incubation program, with the temperature set at 100°F and relative humidity set between 55-60%. After ED18, each tray was removed from the setter and all eggs were candled to discard infertile eggs. After candling, the HH fertile eggs were placed back per pen in Petersime hatcher baskets in the hatcher until ED 21 with a temperature of 99.5°F descending to 99.1°F and a relative humidity of 90% descending to 85% for the last 8 hours. On the first day of life (D0/ ED21), all HH baskets were taken out of the hatcher and all chicks were individually marked and chick quality was assessed (described below, in section *Measurements*). All HH chicks were transferred within 10 min into the barn and allocated to the pens.

**Figure 1:**
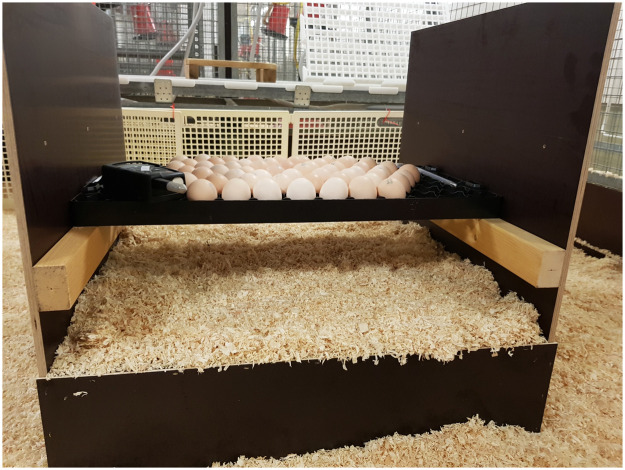
Small “prototype” of the X-Treck system consisting of a wooden frame with two open and two closed sides.

After candling the OH eggs at ED18, fertile eggs were transferred to the experimental barn in a tray (HatchTech 88, HatchTech, De Klomp, The Netherlands) and placed in small “prototype” of the X-Treck system (Fig. 1; Vencomatic, Eersel, The Netherlands). The OH eggs were placed in the pens in which the animals hatched and reared later. The transport time from the hatchery to the barn was maximum 10 min. During transport, eggs were protected from the outdoor climate with blankets. The prototype consisted of a wooden frame with two open and two closed sides. There were two levels between the closed walls: the first level was 0.16 m above the bedding surfaces. This level was closed and littered with wood shavings. The second level contained the egg tray at a height of 0.42 m. The tray was designed to allow hatchlings to fall through the tray holes onto the intermediate littered level. This level served as a resting place for the hatchlings and when they were dry and active, they could explore the littered stable floor, where feed and water were provided.

The barn climate was automatically regulated to a temperature (T) of 94°F and relative humidity (RH) ranged between 40 to 70%. Between ED18.5 and 19.5 T and RH were adjusted depending on eggshell temperature measurements, which were checked every 4 to 6 hours. The target temperature of the eggshell temperatures was between 35 and 38°C. To control the relative humidity in the barn, six additional diffusors were installed. From ED18 to D0 the light was constantly on. After hatching, the X-Treck “prototype” with unhatched eggs was removed from the pens and unhatched eggs were evaluated.

For both treatments, 2^nd^ grade chicks were assessed according to the procedure described by Lourens et al. (2005) and taken out per pen. Second-grade chicks were animals that showed the following characteristics: abnormal (e.g., cross beak, additional legs, club down, short down), small chickens in combination colored pale, pour navel (black dot larger than 0.5 cm and is visible), yolk outside the body, extreme red hocks, green colored chicken, splayed legs, leg issues (unable to stand), bleeding, stargazer, and other abnormalities.

*Animals and Equipment.* After the hatching procedures, a total of 1,600 chickens were reared in experimental pens in three trials (1^st^ and 2^nd^ trial 600 chickens each – 12 pens per trial = 6 repetitions per treatment combination, 3^rd^ trial 400 chickens, 8 pens per trial = 2 repetitions per treatment combination, Tab. 1). All pens had a size of 6 m^2^ (L (length) x W (width): 2 × 3 m) and were littered with wood shavings. Feed (3-phase-pelletized feed: starter D0-D6, 21.6% crude protein, 12.6MJ ME/kg; grower D7-D27, 20.5% crude protein, 13 MJ ME/kg; finisher D28-D35, 19.5 % crude protein, 13.2 MJ ME/kg) was offered *ad libitum* by two feeding troughs and water was provided by round nipple drinkers (8 nipples/pen). From D0 to D3, the light (minimum 20 lx) were on for 23 h. From D4 to D7, the light was reduced stepwise16 h per day. The dark phase including dimming for 15 min between light and dark period and vice versa.

**Table 1:** Overview of the distribution of the four treatments (Hatching system: HH – hatchery-hatched chickens, OH – on-farm hatched chickens; Environment: control – without elevated platforms, enriched – with elevated platform) of the pens and repetition number during the entire rearing periods of three trial (one pen represents one repetition)

	Hatching system*environment				Pens in total
	OH*enriched	OH*control	HH*enriched	HH*control	
Trial 1	3 pens	3 pens	3 pens	3 pens	12 pens
Trial 2	3 pens	3 pens	3 pens	3 pens	12 pens
Trial 3	2 pens	2 pens	2 pens	2 pens	8 pens
Repetitions in total	8	8	8	8	

The enriched pens were equipped with elevated platforms (L x W x H (height): 2 × 0.6 × 0.5 m; space available for about 65% of the chickens) from the first day onwards. The surface and the associated ramp (L x W: 0.9 × 0.6 m; inclination angle of 29.1°) were made of plastic grids (mesh size, 18.7 × 20 mm; slat width, 10 mm; broiler grids, MIK International GmbH, Ransbach-Baumbach, Germany). The area under the elevated platform was not accessible for the chickens. The control groups that were kept without elevated platform were offered the same space, so total floor space was equal to the enriched pens.

### Measurements

*Hatchability and Day-old Chick Quality.* Hatchability was calculated as a percentage per pen by dividing the number of first grade chickens by the alive embryos at ED18. At D0, all chicks that were placed in the pens were assessed for the following indicators: sex, body weight, chick length, and navel, beak and hock condition. Hatchery-hatched (HH) chicks were taken out of the breeder at ED21, i.e. the expected average hatch day. Nevertheless, already from ED19 onwards, chicks can start hatching, which means that until ED21 chicks remain in the brooder without food and water. OH chicks have at any hatch day food and water available in the barn. We consider E21 as day of hatching for the HH hatched chickens, as a more precise date was technically not available.

First, the sex was determined by feather sexing. Males and females (to the same proportion: 50:50) were wing tagged and placed separately in small boxes per pen for further assessment. Then, each individual chick was weighed and chick length was measured with a ruler by stretching the chick apart from the tip of the beak to the tip of the middle toe (excluding the nail; Hill, 2001). Third, the navel was scored using a 3-point scale by Molenaar et al. (2010): 1 = good (closed and clean), 2 = moderate (small black dot or black threat), and 3 = poor (open navel or black dot larger than 2 mm). Beak and hocks were assessed with a 2-point scale: 0 = no red beak or hocks, and 1 = red beak or hocks.

Performance and Welfare Indicators. During the daily checks, chickens, feed and drinkers were inspected, and dead or weak chickens were removed and recorded. Mortality rate per pen was calculated using the initial number of animals and the final number of animals. All chickens were weighed at the beginning (D0) and end (D35) of the rearing period and 10 chickens per pen were randomly selected and weighed during the rearing period at the beginning of every week of age (D7, D14, D21, D28). Additionally, feed intake was calculated per pen by weighing the feeding troughs for each feeding phase. Feed conversion rate was calculated for each phase and the entire rearing period and corrected for chickens that died. In addition, cloaca temperature was measured with a thermometer (Microlife® VT 1831, range 32.0 to 42.9°C, Microlife AG Swiss Corporation, Wildnau, Switzerland) every day during the first week of age of one male and one female chicken from each pen and from the second week of age onwards once a week.

At the end of the rearing period (D35), 10 random chickens of each pen (total of 80 chickens per treatment) were assessed for plumage cleanliness and footpad and hock quality according to the Welfare Quality® (2009) scoring system. State of plumage cleanliness of the chest was scored using a 4-point system: 0 = clear and fluffy, 1 = slightly dirt, 2 = moderate dirt, and 3 completely dirty. Footpad dermatitis and hock burns were assessed according to a 5-point system: 0 = no evidence of dermatitis or hock burn, 1 = slight changes, 2 = moderate changes, 3 = major changes, 4 = severe indication of foot pad or hock burn.

*Behavior.* From the first day of age onwards, each pen was equipped with bird's-eye infrared video camera (Model VTC-E220IRP, color camera for corner mount with IR-LEDs and a fixed local length of 2.9 mm; SANTEC BW AG, Ahrensburg, Germany), which allowed the entire pen to be recorded. The cameras were connected to a PC that recorded and stored the videos as avi-files. In addition to this format, the PC calculated with a self-customized algorithm the brightness differences of the video frames one time in every second and stored these results on the same external hard drive in a txt-file (values of the brightness differences). The work steps of the self-customized algorithm are as follows: On the camera image (frame), the animals have a different brightness compared to the background. If the animals have moved across the ground in one second, there is a difference in brightness in the areas from frame to frame. The more an animal moves, or the more animals move, the greater the difference in brightness. The difference in brightness is therefore used as an indicator of movement.

For the analysis of usage of elevated platforms per pen, the recorded video data from the section “Group activity” was used. By counting the number of chickens on the elevated platforms using scan sampling with 1 h intervals over 24 h for two successive days for each week of age, the percentage of use was determined per pen.

To evaluate the walking ability, a rotarod test modified for chickens was performed (Malchow et al., 2019c). This test measures the physical ability to regulate body balance (Hamm et al., 1994). The apparatus was set up in the same barn at the end of the pens in an open area. To ensure that the tested animals could not visually perceive other animals, visual blockades in the form of boards were installed on both sides of the frame. For the test, a single chicken was placed on a rod 85 cm high, initially stationary and gradually rotating faster. The duration (“latency to leave”) how long a chicken remained on the rotating rod was recorded. To minimize the risk of injury to chickens leaving the rod in an uncontrolled manner, soft mats were placed over a large area at a distance of 20 cm below the rod. For each pen and treatment, two female and two male chickens were tested.

Three different behavioral tests were used to test fearfulness: arousal test, and open-field test. The arousal test according to Phi van et al. (2018) was performed at D5 with 10 chickens per pen and treatment (n= 320 chickens in total). A mobile test arena (L x W x H, 1 × 1 × 0.5 m) with 25 squares of equal size (L x W, 20 × 20 cm) drawn on the floor was placed in each home pen. The randomly selected animal was carefully placed in the center of the test arena (olfactory as well as acoustic contact with conspecific was maintained). The chickens remained in the test arena for three minutes. During this time, the latency until the chickens first left the central square, the frequency of crossing the squares and the frequency of defection were recorded by direct observation. The observer sat directly next to the test arena. After the test, the chicken was placed back in the pen.

In the fourth week of age, an open-field test was carried out, whereby the same test arena as in the arousal test was placed outside the pens at the end of the stable corridor. The same chickens were tested as for the arousal test. The chickens were taken out of the pen and transferred to the test arena. The chickens were placed in the center of the test arena. The same parameters as in the arousal test were recorded within five minutes.

### Statistical Analysis

*Hatchability and Day-old Chick Quality*. Hatchability was analyzed with a linear mixed effect model (LME) with hatching system (2 factor level: HH *vs.* OH) as explanatory factor and nested random factors Pen within trial. Hatchability was arcsin-transformed (arcsin(sqrt(x)) to obtain a normal distribution of the residuals.

With a similar structured LME, body weight at hatching was analyzed after log(x+1) transformation. Chick length at hatching (day 0) was analyzed using a LME model with hatching system, sex and their two-way interaction as explanatory factors. A nested random factor of Pen within trial was included. Navel condition was analyzed using a general linear mixed effect model (GLMM) with Poisson distribution with the above-mentioned factors and random effects.

*Performance.* Mortality between groups were compared using a Gehans-Wilcox Test with factors hatching system and environment using the package “survival” (Therneau, 2023; Therneau and Grambsch, 2000). Live body weight between day 7 and 35 was analyzed with a LME with hatching system (HH *vs.* OH), environment (enriched *vs*. control) and day of life (7,14,21,28,35), sex (male, female) as well as their two-way interactions as explanatory factors. As nested random effects Animal-ID within Pen within trial were taken into account. Body weight was log(x+1) transformed to achieve normal distributed residuals. Body temperature between day 0 and 35 was analyzed with a LME with hatching system (HH *vs.* OH), environment (enriched *vs.* control) and day of life (0,1,2,3,4,5,6,7,14,21,28,35), sex (male, female) as well as their two-way interactions as explanatory factors and additionally time of day. As nested random effect Animal-ID within Pen within trial were taken into account.

Footpad scores and hock burn scores were analyzed each with a GLMM with binomial distribution (as only “0” and “1” scores were obtained) and with hatching system, environment and their two-way interaction as explanatory factors. Pen within trial was considered as nested random factor. For plumage cleanliness a similar model was calculated but with a Poisson distribution.

*Behavior.* Group Activity, measured as the change of brightness in the video frame stream, was analyzed using a LME with environment, hatching system, day (1-7) and their respective two-way interactions as explanatory factors and week within Pen within trail as random nested factors.

Usage of elevated platform (for each hour: number animals on platform/number of all animals in the pen) was analyzed in separate LME models for light- and dark-phase both with the explanatory factors hatching treatment, age of life and their interaction. As nested random factor daytime, within day, within week, within Pen and trial were considered.

Walking ability latencies, i.e. the times birds needed to jump off the rotating rod, were analyzed using a LME with hatching systems, environment, sex and their two-way interactions as explanatory factors. As random nesting factors trial and Pen were considered. Latencies have been log (x+1) transformed.

In the arousal test (fearfulness test) at day 5 the latency to make a first field change as well as the activity (fields changed/(180-latency)) were analyzed each with a LME with hatching system, environment, sex and their two-way interactions as explanatory factors and Pen within trial as random nested factor. Latency to first field change and activity were log(x+1) transformed. In the Open Field Test (fearfulness test) conducted at the days 25, 26 and 27 of life the latency to make a first field change as well as the activity (fields changed/(300-latency)) were analyzed each with a LME with hatching system, environment, sex and their two-way interactions as well as day of life (25,26,27) as explanatory factors and Pen within trial as random nested factor. Latency to first field change was log(x+1) transformed.

All tests were calculated in R 4.3.0 (R Core Team, 2023) using the package “nlme” (Pinheiro et al. 2023; Pinheiro and Bates, 2000) for LME. Some LME showed minor deviations from assumptions but LME are assumed to be robust against these (Schielzeth et al. 2020). “lme4” (Bates et al., 2015) and “car” (Fox and Weisberg, 2019) were used to calculate GLMM and the respective p-values.

## RESULTS

### Hatchability and Day-old Chick Quality

The hatchability did not differ significantly between the OH treatment (mean ± SD 96.5 % ± 3.37%) and the HH treatment (96.5% ± 2.76%) (LME, factor hatching system F_1,28_=0.008, p=0.93). Live body weight at hatch was significantly affected by an interaction between hatching system and sex (LME, factor hatching system F_1,28_=79.14, p<0.0001; factor sex F_1,1566_=0.28, p=0.60; factor hatching system*sex F_1,1566_=12.37, p=0.0004; Tab. 2). OH chicks were heavier than HH chicks and in OH female were heavier than males, while in HH males were heavier than females. Chick length at hatching was significantly affected by hatching system and sex, but no interaction was found (LME, factor hatching system F_1,28_=7.31, p=0.02; factor sex F_1,1264_=10.72, p=0.001; factor interaction sex*hatching system F_1,1564_=0.02, p=0.89). Male chickens had a shorter body length (19.4 cm ± 0.7 S.D.) than female chickens (19.5 cm ± 0.6 S.D.) and OH chickens were shorter (19.4 cm ± 0.7 S.D.) than HH chickens (19.6 cm ± 0.6 S.D.).

**Table 2:** Live body weight (± S.D.) at day 1, 7, 14, 21, 28, 35 of chickens hatched on-farm and conventional with sex. Significant interaction between hatching system and sex represented by different letters for the first week of life (p<0.01).

Day of age		mean OH [g]	S.D. [g]	mean HH [g]	S.D. [g]
1	female	46.7a	4.8	40.9c	2.8
	male	46.1a	4.7	41.3b	3.0
7	female	199.8	18.6	185.4	13.2
	male	202.5	18.5	189.7	17.2
14	female	497.7	60.9	473.6	36.2
	male	533.7	64.5	503.3	44.3
21	female	980.4	74.6	939	78.5
	male	1084	132	1040.7	99.5
28	female	1592.2	123.4	1544.4	127.9
	male	1835.3	181.6	1806.5	138.4
35	female	2264.9	203.7	2230.1	198.3
	male	2610.1	271.4	2616.4	248

Navel condition did not differ between hatching system or sex (GLMM, factor hatching system df=1, χ^2^=0.22, p=0.64, factor sex df=1, χ^2^=0.13, p=0.71, factor hatching system*sex df=1, χ^2^=0.03, p=0.86).

Red beaks were scored with “1” in nine out of 1,600 animals being scored in total (all from conventional hatchings system) and because of low variation a statistical analysis was omitted. For red hocks, eleven animals were scored with “1” (five from conventional- and 6 from on-farm hatching, and with respect to environment 6 from control and 5 from enriched).

### Performance

Mortality in the first week was neither different between hatching systems nor between post-hatch environment (Gehans-Wilcoxon Test χ^2^=1.9, df=3, p=0.60, Tab. 3). Mortality during the rearing period was neither different between hatching system nor between environment (Gehans-Wilcoxon Test χ^2^=0.3, df=3, p=1.00, Tab. 2).

**Table 3:** Mortality during the first week of life and during the entire rearing period of chickens hatched in different hatching systems and post-hatch enrichment (Hatching system: HH – hatchery-hatched chickens, OH – on-farm hatched chickens; Environment: control – without elevated platforms, enriched – with elevated platform; all p≥0.60)

Hatching system * Environment	Mortality 1^st^ week of life [%]	Mortality entire rearing period [%]
HH * Control	0.5 ± 1.4	2.5 ± 2.1
HH * Enriched	0.3 ± 0.7	2.0 ± 1.5
OH * Control	0.5 ± 0.9	2.8 ± 2.6
OH * Enriched	1.0 ± 1.5	2.5 ± 2.1

Live body weight over the rearing period was affected by hatching system, age, sex as well as by environment (LME, factor hatching system, F_1,28_=12.50. p=0.001; factor environment F_1,1256_=38.62, p<0.0001; factor day of life F_1,1256_=70934.93, p<0.0001; factor sex: F_1,1538_=296.15, p<0.0001; factor hatching system*environment F_1,1256_=0.18, p=0.67; factor hatching system*day of life F_1,1256_=12.57, p=0.0004; factor environment*day of life F_1,1256_=2.41, p=0.12; factor hatching system*sex F_1,1538_=0.20, p=0.66; factor environment*sex F_1,1256_=1.21, p=0.27; factor day of life*sex F_1,1256_=63.08, p<0.0001; Tab. 2; Fig. S1; Tab. S1). Live body weight increased with age, OH birds were heavier than HH and males were heavier than females during the entire rearing period. Furthermore, chicks from control groups were slightly but significantly heavier than chicks with elevated platforms throughout rearing. Table S5 shows the feed conversion rate descriptively.

**Figure 2:**
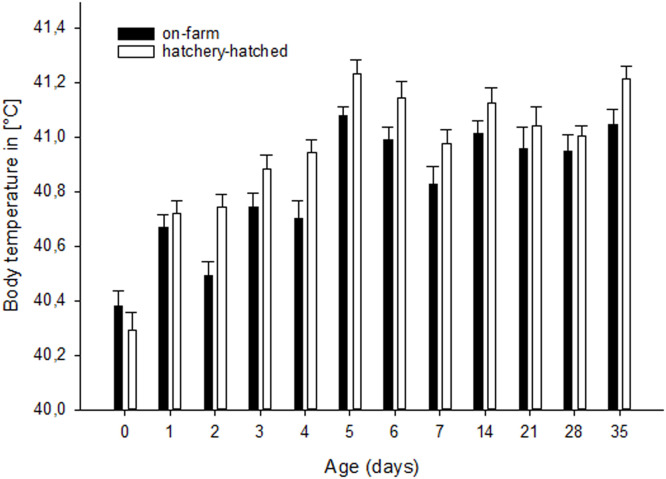
Body temperature (± S.E.) throughout rearing period from day 0 to day 35 of life of HH (hatchery-hatched) and OH (on-farm hatching) chickens. The hatching system had a significant effect on body temperature (p=0.002), as well as day of life (p<0.0001).

Body temperature was significantly different between hatching system and between age. Body temperature increased by age and was higher in the HH treatment. (LME: factor hatching system, F_1,15_=14.0, p=0.002, factor environment F_1,15_=0, p=0.54, factor sex F_1,17_=0.0, p=0.60, factor day of life F_11,395_=43.0, p<0.0001; factor daytime F_1,395_=1, p=0.24, factor hatching system*environment F_1,15_=0.0, p=0.7, factor hatching system*sex F_1,17_=0.63, factor hatching system*day of life F_11,395_=2.0, p=0.10, factor environment*sex F_1,17_=0.0, p=0.54, factor environment*day of life F_11,395_=1.0, p=0.34, factor sex*day of life F_11,395_=1.0, p=0.75; Fig. 2; Tab. S2).

Plumage cleanliness at d32 of age was also not different with respect to hatching system or environment (GLMM, factor hatching system df=1, χ^2^=0.22, p=0.64; factor environment df=1, χ^2^=1.27, p=0.26; factor hatching system*environment df=1, χ^2^=0.00, p=0.95, Tab. 4).

**Table 4:** Proportions of broiler chickens at the end of the rearing period showing the respective scores for plumage cleanliness, footpad dermatitis and hock burn (Hatching system: HH – hatchery-hatched chickens, OH – on-farm hatched chickens; Environment: control – without elevated platforms, enriched – with elevated platform; Scoring of plumage cleanliness: S0 = clear and fluffy, S3 = completely dirty; Score of footpad and hock burns: S0 = no evidence of changes, S4 = severe indication; n/treatment = 80)

Hatching system * Environment	Plumage cleanliness [%]				Footpad dermatitis scores [%]					Hock burn scores [%]				
	S0	S1	S2	S3	S0	S1	S2	S3	S4	S0	S1	S2	S3	S4
HH * control	14	68	19	0	99	1	0	0	0	98	3	0	0	0
HH * enriched	28	58	15	0	100	0	0	0	0	96	4	0	0	0
OH * control	14	75	11	0	99	1	0	0	0	100	0	0	0	0
OH * enriched	31	58	11	0	100	0	0	0	0	100	0	0	0	0

Footpad dermatitis scores at d35 of age were not different with respect to hatching system or environment (GLMM, factor hatching system df=1, χ^2^=0, p=0.99; factor environment df=1, χ^2^=0, p=0.99; factor hatching system*environment df=1, χ^2^=0, p=0.99, Tab. 2).

Hock burns at d32 of age did not differ between hatching system or environment (GLMM, factor hatching system df=1, χ2=0, p=0.99; factor environment df=1, χ2=0.14, p=0.70; factor hatching system*environment df=1, χ2=0, p=0.1, Tab. 4).

### Behavior

**Figure 3:**
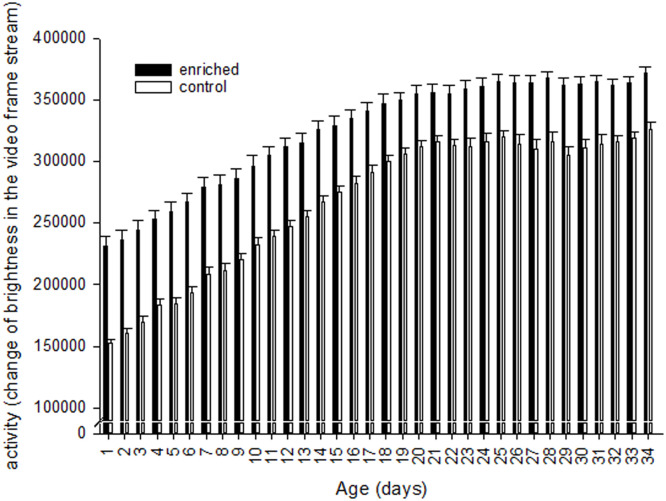
Mean group activity (± S.E.) throughout rearing period from day 1 to day 34 in enriched and control pens. (Environment: control – without elevated platforms, enriched – with elevated platform)

The activity on group level based on video image analysis was affected by environment and age but not by hatching system (LME: factor hatching system F_1,26_=0.9, p=0.35; factor environment F_1,26_=39.1, p<0.0001; factor day F_6,909_=128.8, p<0.0001; factor environment*hatching system F_1,26_=2.4, p=0.13; factor hatching system*day F_6,909_=0.2, p=0.98; factor environment*day F_6,909_=7.0, p<0.0001, Fig. 3, Tab. S3). The group activity was higher in groups with elevated platforms compared to the control treatment group.

**Figure 4:**
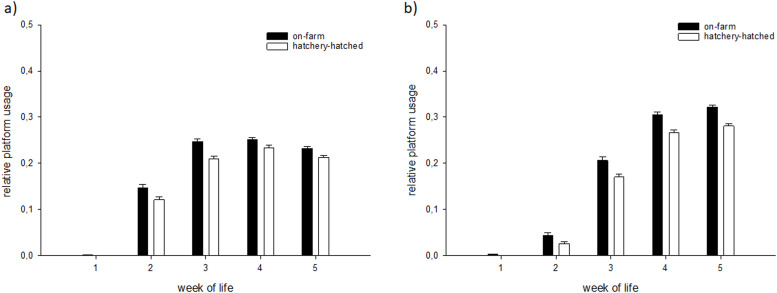
Mean relative usage (± S.E.) of platforms for OH chickens (on-farm) or HH chickens (hatchery-hatched); a) the light phase and b) the dark phase.

The usage of elevated platforms in the respective pens during the light-phase was affected by age but not by hatching system or the interaction (LME, factor hatching system F_1,12_=2.59, p=0.13; factor week of life F_1,62_=151.77, p<0.0001; factor hatching system*week of life F_1,62_=0.10, p=0.76; Fig. 4a, Tab. S4). The usage of elevated platforms during the dark-phase was affected by age and tended to differ between hatching system treatments (LME, factor hatching system F_1,12_=3.82, p=0.07; factor week of age F_1,62_=502.22, p<0.0001; factor hatching system*week of age F_1,62_=1.62, p=0.21; Fig. 4b).

The walking ability as measured in a rotarod test was neither affected by environment nor by hatching system but differed between sexes (LME: factor hatching system F_1,26_=1.47, p=0.24; factor environment F_1,26_=0.06, p=0.82; factor sex F_1,98_=4.74, p=0.03; factor hatching system*environment F_1,26_=1.43, p=0.24; factor hatching system*sex F_1,98_=0.20, p=0.66; factor environment*sex F_1,98_=2.60, p=0.11). Females remained longer on the rotating rod, whereby it is important to mention that all latencies were relatively short (2.9 s *vs.* 3.6 s for males and females respectively).

**Figure 5:**
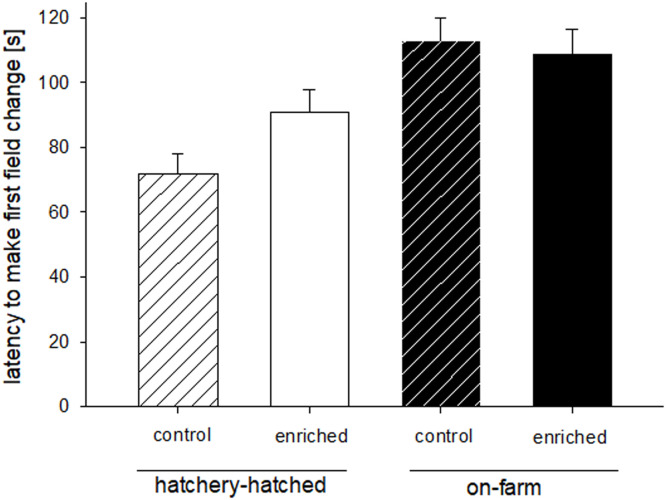
Latency to the first field change ([s]; mean ± S.E.) of chicks in the arousal test from hatchery-hatched chickens or on-farm chickens and were reared either under control (without elevated platforms) or under enriched (with elevated platforms) conditions (p=0.005).Supplement Material

In the arousal test at day 5 of age, a significant effect was found of hatching system and sex on the latency to make the first field change in the test (LME: factor hatching system F_1,26_=9.29, p=0.005; factor environment F_1,26_=0.001, p=0.97; factor sex F_1,285_=7.23, p=0.008; factor hatching system*environment F_1,26_=1.51, p=0.23; factor hatching system*sex F_1,285_=0.68, p=0.42, factor environment*sex F_1,285_=2.65, p=0.10; Fig. 5). HH chickens were faster in making a first field change compared to OH chickens (Fig. 6). Furthermore, females were faster (86.38 s ± 63.86s) than males (105.83s ± 63.92s) in making a first field change. Activity was not significantly affected by any of the factors (LME: factor hatching system F_1,26_=1.32, p=0.26; factor environment F_1,26_=2.67, p=0.11; factor sex F_1,188_=1.42, p=0.24; factor hatching system*environment F_1,26_=3.87, p=0.06; factor hatching system*sex F_1,188_=0.14, p=0.71, factor environment*sex F_1,188_=1.67, p=0.20).

The latency in the Open Field test conducted between day 25-27 was not affected by any of the factors (LME: factor hatching system F_1,62_=0.47, p=0.50; factor environment F_1,26_=1.01, p=0.32; factor sex F_1,286_=2.35, p=0.13; factor day of life F_1,286_=2.95, p=0.09; factor hatching system*environment F_1,26_=0.09, p=0.77; factor hatching system*sex F_1,286_=2.70, p=0.10; factor environment*sex F_1,286_=0.46, p=0.50). Activity in the Open Field test was affected by environment and sex (LME: factor hatching system F_1,26_=0.53, p=0.47; factor environment F_1,26_=7.78, p=0.01; factor sex F_1,248_=17.79, p<0.0001; factor day of life F_1,248_=0.17, p=0.68; factor hatching system *environment F_1,26_=0.006, p=0.94; factor hatching system*sex F_1,248_=0.42, p=0.52; factor environment*sex F_1,248_=6.71, p=0.01). Birds from enriched group were more active (0.0481 ± 0.0386 S.D.) in the Open Field test than birds from control pens (0.0371 ± 0.0294). Furthermore, females (0.0505 ± 0.0393) were more active than males (0.0340 ± 0.0262).

Effects of all results are summarized in Table 5.

**Table 5:** Overview of all parameters regarding hatching system and environment (n.s. = not significant; “>” indicated significant differences between the treatments; striped boxes were not included in the analysis)

	Parameter	Hatching system	Environment	Hatching system * environment	Age	Sex
Hatching	Hatchability	n.s.				
Chick quality	Body Weight	OH > HH				Males > females
	Chick Length	HH > OH				Females > males
	Navel condition	n.s.				n.s.
	Beak condition	n.s.				n.s.
	Hock condition	n.s.				n.s.
Performance	1^st^ week mortality	n.s.	n.s.	n.s.		
	Total mortality	n.s.	n.s.	n.s.		
	Body weight	OH > HH	Control>enriched	n.s.	p<0.0001	Males > females
	Body temperature	HH > OH	n.s.	n.s.	p<0.0001	n.s.
	Plumage	n.s.	n.s.	n.s.		
	Footpad	n.s.	n.s.	n.s.		
	Hock burn	n.s.	n.s.	n.s.		
Behavior	Activity	n.s.	Enriched> control	n.s.	p<0.0001	
	Usage platform	Light: n.s., dark: p=0.07 (OH > HH)			p<0.0001	
	Walking ability	n.s.	n.s.	n.s.		Females > males
	Arousal (latency)	HH > OH	n.s.	n.s.		Females > males
	Arousal (activity)	n.s.	n.s.	n.s.		n.s.
	Open field	n.s.	n.s.	n.s.		n.s.

## DISCUSSION

We expected that the combined treatment of hatching system and environment would affect different parameters of performance and behavior and could improve animal welfare in broiler chickens. In summary, there was no interaction between environment and hatching system but each factor on itself and sex influenced different parameters related to performance and animal behaviour, as summarized in Table 5.

### Hatching and Day-old Chick Quality

We showed that the hatching rate (ED18 to ED21) did not differ between the two hatching systems and this indicated that the hatching environment did not have an effect on the survival rate of the chicken embryos. Although environmental settings of for example temperature, relative humidity, CO_2_ concentration and air speed were different between the two hatching systems, the purpose in both systems was to maintain the eggshell temperature within a range of 96 to 100°F from ED18 until the start of hatching process. Larger deviations from this EST range can negatively affect hatchability, as shown in previous studies (Lourens et al., 2005).

Other studies such as Souza da Silva et al. (2021) or de Jong et al. (2019) found as well that hatchability was not affected in both chickens coming from young and prime broiler parent flock when using OH or HH systems. Furthermore, we assumed that the transportation (less than 10 min) of the incubated eggs on the ED18 had no negative influence on the hatching rate. Embryonic mortality is further influenced by the age of the parental flock, and mortality is increasing with increasing age of the parental flock (Almeida et al., 2008). In the current study, eggs from a young parent flock (27 to 32 weeks of life) were used in all three trials and they hatched alike conventional.

The OH chicks had a higher body weight compared to the HH chicks at D0. Studies by de Jong et al. (2019), and Souza da Silva et al. (2021) showed as well that OH chickens were heavier than the HH chickens, which is probably mainly explained by the earlier access to feed and water directly after hatch in the OH chickens. The HH chickens had no supply of feed and water, except the amount of the yolk sac (Bergoug et al., 2013), and had to wait until they arrived at the barn (time ranging between 2 and 40 h in the current study, depending on hatching moment). Accordingly, the first OH chickens probably had up to 40h more time to consume feed and water compared to the HH chickens which can contributed to the higher body weight of OH chicken on D0. Additionally, males were already heavier than females on the first day after hatch. It is known that weight development of males and females differs right from the beginning (Mesquita et al., 2021) and males reach a higher body weight due to their higher growth rate (López et al., 2011).

In our study, HH chicks had a longer chick length than OH chicks and females were longer than males on the first day of life. In parallel with the higher body weight in OH chickens, we expected that the OH chicks would also be longer as was the case in a study by Souza da Silva et al. (2021). However, also de Jong et al. (2020) found that HH chickens tended to be longer than OH chicks and the reason for this discrepancy between studies is unknown as in all of these studies the chickens that received immediately feed and water post-hatch were heavier. Molenaar et al. (2008) showed, opposite to our results, that males were longer than females at hatch but found similarly that males showed a higher live body weight at the end of fattening than females. This indicates a relationship between chick length and post-hatch body weight (Willemsen et al., 2008). Differences may be due to differences in strain or age of the breeder flock. Taking these results together for our study, the chick length measurements do not seem to be related to body weight differences found in day-old chicks by early feed treatment and related to performance of the chickens in the further rearing period, which was also found by Willemsen et al. (2008).

The prevalences of abnormalities of the navel, beak or hock were very low and, thus, we did not find differences between the hatching systems or sexes or it was not possible to be analyzed. This is in contrast to other studies where poorer navel scores and more red beaks were found in the OH compared to the HH treatment group (de Jong et al., 2019; de Jong et al., 2020; Souza da Silva et al., 2021; Jessen et al., 2021b) possibly indicating that a higher variability of environmental conditions during on-farm hatching (e.g. regarding ambient temperature and humidity levels) may negatively affect these parameters, but this may not have been the case in our study where relatively small experimental rooms were used. Additionally, it may show that the hatching process for both hatching systems did not negatively affected chick quality. In summary, it was found that OH treatment did not affect hatchability and increased bodyweight of day-old chicks but no other chick quality parameters were affected.

### Performance

The mortality in total and the first-week mortality did not differ between treatment groups in our study. Some studies related to OH found the same results as in our study (de Jong et al., 2019; Souza da Silva et al., 2021), but other studies found also lower mortality of OH chickens (de Jong et al., 2020). Likewise for body weight development, it can be hypothesized that mortality is lower in OH chickens due to earlier feed and water access (de Jong et al., 2017) and reduction of other stressors such as processing and transport. It seems that transport (Mitchell, 2009, Yerpes et al., 2021) and related procedures such as handling of newly hatched chicks (Knowles et al., 2004) can have an impact on their survival rate and development in later life. In our study, the chicks were manually removed from the incubator and gently handled to assess chick quality which may result in a much lower stress level than in a commercial hatchery where chickens are taken out of the hatchers, processed over several conveyor belts, subjected to the chick counter, often spray vaccinated and placed in the chick storage room before transportation (Hedlund et al., 2019). The processes in the current study took max. 2h after opening the incubator. There was no assembly line on which the chicks were moved. In addition, the HH chicks in our study did not have a long transport duration from the incubator to the barn (max. 10 min), which excluded very long feed deprivation times. Another reason for a similar mortality could be the selection of the 2^nd^ grade chicks which have been sorted out carefully for both treatments by the same experienced staff. This differs in practise where personnel of either the hatchery or the farm perform the assessment.

Sex (males were heavier than females), hatching system (OH chickens were heavier than HH chickens), and environment (control groups were heavier than groups with elevated platforms) influenced the live body weight during the rearing period. Various studies have shown that fast-growing broiler males reach a higher weight than females (Petek and Orman, 2013, de Almeida et al., 2017; Hammemi et al., 2024) due to the fact that males have a higher growth rate (de Almeida et al., 2017). Our study showed the same result. The enrichment of the environment with elevated structures (perches or platforms) had either no effect (Ventura et al., 2010; Malchow et al., 2019b; Spieß et al., 2022) or a negative effect on body weight (Güz et al., 2021) when compared to animals without elevated structures. In our study, the elevated platforms as enrichment showed a negative effect on body weight (i.e. lower weight), which could be explained by the higher activity and usage of elevated platforms. OH chickens showed a higher weight during the rearing period compared to HH chickens, confirming the results of other studies (Giersberg et al., 2021, Guyot et al., 2023), except for the study by de Jong et al. (2019), in which no difference in weight was shown. Due to the early intake of feed and water and already higher body weight on the first day of life, the animals had a better development at the start, which was reflected in their weight over the entire rearing period.

Hatching system and age affected rectal body temperature. HH chickens had a higher body temperature of 1°C compared to OH chickens over the entire rearing period which does not seem to be related with the difference in live weight between the treatment groups. A point with regard to body temperature is that heavier animals generally have a higher temperature than lighter animals (Skinner-Noble and Teeter, 2004). In our study, however, the OH were heavier, but the HH animals showed a higher body temperature over the entire rearing period. Furthermore, body temperature increased by age and differed around 1°C between weeks of life. The lower body temperature of OH chickens may be related to more fluctuating eggshell temperatures and thereby embryo temperatures during on-farm hatching compared to the hatcher as has been shown previously (Molenaar et al., 2023). If the embryos experienced fluctuating temperatures during the hatching process, this may have had an effect on post-hatch body temperature (Collin et al., 2005, Yalcin et al., 2022), see also Morita et al. (2016) during the first post-hatch days. Another difference in the environmental conditions from ED18-21 onwards was the lighting between the treatment groups. There was no light in the incubator and the lights were on for 24 h a day in the barn. It has been reported that lighting during the last part of the embryonic phase can affect the circadian cycle of body temperature after hatching (Hill et al., 2004), whereby constant lighting of the eggs can lead to lower body temperature in posthatch animals compared to animals that have received regular dark periods during the incubation period. Furthermore, the average body temperature of the chickens from our study was higher, but in a normal range and not above 42°C compared to another study using a pure broiler line breed (Erensoy et al., 2024), or Hamrita and Conway (2017) and Piestun et al. (2013). The body temperature was always measured in the morning, with the body temperature being higher in the morning than in the afternoon or evening (Hill et al., 2004). Another explanation for the higher temperature could be the handling during the measurement process, as this causes stress, which can also cause the body temperature to rise (Iyasere et al., 2017). A lower body temperature could suggest a better adaptation to higher ambient temperatures in later life (= higher thermoneutral zone (Morillo et al., 2023)), which could give the OH chickens an advantage during heat stress conditions.

The findings on plumage cleanliness, footpad dermatitis and hock burns were without or only with small abnormalities for all broiler chickens. There were no differences between the treatment groups. In another study, the provision of perches in the housing environment had positive effects on footpad quality (Hongchao et al., 2014), but offering perches and platforms also showed no positive or even a negative influence on plumage cleanliness (Malchow et al., 2019a). In addition, the OH chickens showed better foot pad quality in the study of de Jong et al. (2019) and Giersberg et al. (2021). All three parameters (plumage cleanliness, footpad dermatitis, and hock burn) can depend on the quality of the litter (Jessen et al., 2021b, van der Eijk et al., 2023). In our case under experimental conditions, new substrate was added from the third week onwards if it was too moist, which resulted in a good litter quality and, thus, a reduction of possible effects of litter quality on contact dermatitis.

### Behavior

The provision of elevated structures, such as platforms, increased the group activity of the broiler chickens compared to control groups, but hatching system had no influence. In general, during the rearing period the activity decreased from the first to the last day. The groups of broilers studies by Bailie et al. (2013) and Vasdal et al. (2019) also showed an increase in activity due to the provision of environmental enrichment (straw bales and elevated platforms). Surprisingly, we could not find any differences between the hatching systems. In other studies, lower activity was found in OH chickens (Jessen et al., 2021a; Giersberg et al., 2023). Likewise, no differences in activity and of fearfulness later in life were found in the arousal test in our study. Perhaps one reason could be that the differences between HH and OH were comparably small because, for instance, also for HH the transport time was less than 10 min, which could not have influenced fearfulness (Hollemans et al., 2018). Furthermore, a decrease in activity with increasing age independent of the husbandry environment can also be seen in other studies such as the ones by Bizeray et al. (2002), Norring et al. (2016), and Malchow et al. (2018). In general, broiler chickens become more inactive with increasing age and weight (Baxter et al., 2021), and activity can also be reduced by a poorer walking ability with increasing age (Silvera et al., 2017).

In general, the usage of elevated platforms increased in both hatching systems with increasing age. However, there was less use during the light period in the 5th week than in the previous week of life. At night, OH chickens tended to use the elevated platforms more. Malchow et al. (2018) and Schomburg et al. (2023) also showed a similar pattern of use. However, May et al. (2024) showed a decrease in the use of platforms by broilers at night. Up to this point, no study, to our knowledge, has investigated different hatching systems combined with elevated platforms as enrichment. However, Jessen et al. (2021a) were able to show that hatching system did not affect the use of an outdoor run, particularly in slower growing broilers, whereby the outdoor run is seen as an enrichment of the housing environment. The tendency of OH chickens to use the elevated platforms more frequently than HH chickens may be due to the more fearless behavior in the first week (arousal test), resulting in more exploratory behavior (Baxter et al., 2019). It also appeared that the earlier the elevated platforms are offered, the better they are used (personal observation), which should be verified with experimental data. However, in our study, the OH animals did not have earlier access to the elevated platforms than the HH. The structures were offered at the same time.

The treatments did not affect the walking ability of the broilers, only sex had an impact. Female broiler chickens showed better walking ability in the rotarod test than the males. The reason for this difference could be attributed to the differences in body weight and the different weight development, whereby male animals were heavier than females (de Almeida et al., 2017). The same results were also presented by Kestin et al. (1992) and Kestin et al. (2001). However, in those studies gait score was used to measure walking ability. Unfortunately, we cannot make any statement whether female chickens also showed better usage of elevated platforms and higher activity, which in turn could have improved walking ability. Jessen et al. (2021b) and Giersberg et al. (2021) also showed no influence of the hatching system on walking ability.

The results of the arousal test and the open field test did not show the same differences in fear responses for the hatching system and age, as Giersberg et al. (2021) showed. One explanation for the different results of the behavioral tests could be that the motivation to show behaviors was higher in the arousal test because the conspecifics were still in the same environment. In the arousal test in our study, the OH chicks showed a higher fear reaction (longer latency of the first segment change) compared to HH birds. Furthermore, OH chickens from the study by Jessen et al. (2021a) showed less fearfulness, in contrast to Giersberg et al. (2020), where the animals showed increased fearfulness compared to HH chickens in the first week. The two mentioned studies and the current study used different tests for fearfulness. That can also affect the results. An additional assumption for the differences of fearfulness is that animals that hatch in an on-farm environment already achieved better visual development and visual lateralization, which can reduce anxiety and stress, allowing them to cope better in a new environment (Rogers, 2012, Archer and Mensch, 2013). In addition, the female animals showed less anxiety in the arousal test, although Franco et al. (2022) and, Altan et al. (2013) showed no differences between sexes. However, both studies used a tonic immobility test to test fearfulness. We measured fearfulness by the latency to the first segment change according to Faure and Morozeau (1975). Here, another trait can also be discussed, such as the ability to cope with the environment actively or passively (Wechsler, 1999). However, reactive animals show few movement patterns in a new environment (Benus et al., 1991) and female animals tend to show higher movement activity (Balážová and Baranyiová, 2010). Jones (1977) showed that female chickens had a shorter latency to the first step. In general, it seems that hatching system with increasing age no longer affects behavior, as the animals kept in the same environment and thus have the same influencing factors, as well as the genetics are the same.

## CONCLUSION

The interaction of alternative hatching system and elevated platforms did not affect performance or behavior. Hatching on-farm can improve performance in terms of live body weight over the entire rearing period without having negative effects on other commercially relevant traits, such as e.g. hatchability. Furthermore, body temperature of OH chickens was lower than in HH chickens, which could indicate an improvement of the adaptive capacity that facilitates adjustments to different ambient temperatures in later life. In terms of behavior, there was a difference between OH and HH chickens, such as better usage of elevated platforms by OH. Moreover, an increase in activity was found when elevated platform was offered. Overall, both on-farm hatching and enrichment can lead to an improvement of particular performance and behavior measurements and, thus, an improvement of certain aspects of animal welfare.

## Declaration of competing interest

None.

## Appendix Supplementary materials

Image, application 1

Image, application 2
