# Supplementary figures and images for: Effect of on-farm hatching and elevated platforms on behavior and performance in fast-growing broiler chickens
Source: Poult Sci. 2025 Feb 17;104(4):104910. doi: 10.1016/j.psj.2025.104910 (PMC11889558; doi:10.1016/j.psj.2025.104910)

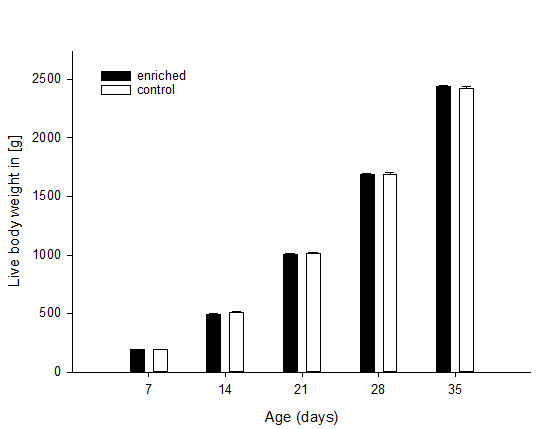

Supplement: Supplementary file 1 [file mmc1.zip › mmc1.tif]
