# Supplementary material for: Effect of on-farm hatching and elevated platforms on behavior and performance in fast-growing broiler chickens
Source: Poult Sci. 2025 Feb 17;104(4):104910. doi: 10.1016/j.psj.2025.104910 (PMC11889558; doi:10.1016/j.psj.2025.104910)
Supplement: Supplementary file 2 [file mmc2.docx]

**Supplement Material**

**Figure**

**
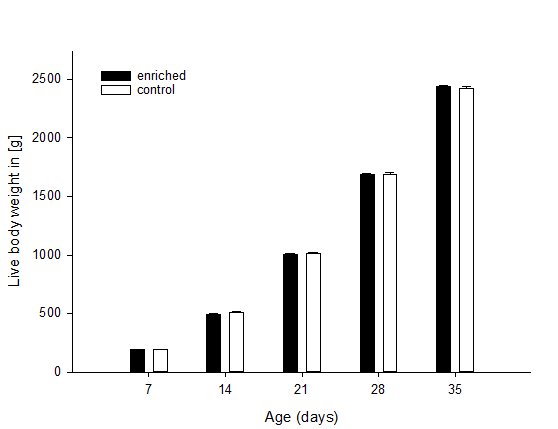
**

**Figure S1** Live body weight (± S.E.) over fattening from day 7 to day 35 of life of chickens kept with (enriched) and without (control) elevated platforms and control conditions.

**Tables**

| **Table S1** Live body weight (± S.D.) at day 1, 7, 14, 21, 28, 35 of chickens hatched on-farm and conventional with environment (control – without elevated platforms, enriched – with elevated platform). | | | | | |
| --- | --- | --- | --- | --- | --- |
|  |  | **HH** | | **OH** | |
| **Age in days** |  | **Mean [g]** | **SD** | **Mean [g]** | **SD** |
| **7** | **control** | 188,0 | 15,5 | 202,1 | 19,8 |
| **7** | **enriched** | 187,3 | 15,8 | 200,4 | 17,8 |
| **14** | **control** | 491,9 | 44,1 | 530,8 | 55,3 |
| **14** | **enriched** | 485,4 | 42,5 | 501,6 | 69,8 |
| **21** | **control** | 987,9 | 108,1 | 1043,6 | 121,6 |
| **21** | **enriched** | 979,2 | 96,0 | 1021,1 | 116,3 |
| **28** | **control** | 1649,8 | 188,5 | 1726,9 | 222,5 |
| **28** | **enriched** | 1674,9 | 185,0 | 1695,4 | 174,4 |
| **35** | **control** | 2419,7 | 300,2 | 2432,4 | 296,0 |
| **35** | **enriched** | 2425,5 | 292,6 | 2445,2 | 295,8 |

| \| **Table S2** Mean (± S.D.) of body temperature [°C] throughout rearing period from day 0 to day 35 of life of HH (hatchery-hatched) and OH (on-farm hatching) chickens in both the control (without elevated platforms) and the enriched environment (with elevated platforms). \| \| \| \| \| \| \| --- \| --- \| --- \| --- \| --- \| --- \| \|  \|  \| **OH** \| \| **HH** \| \| \| **Age in days** \| **Environment** \| **Mean [°C]** \| **SD** \| **Mean [°C]** \| **SD** \| \| **0** \| **control** \| 40,5 \| 0,2 \| 40,4 \| 0,2 \| \| **0** \| **enriched** \| 40,3 \| 0,3 \| 40,2 \| 0,3 \| \| **1** \| **control** \| 40,7 \| 0,1 \| 40,8 \| 0,2 \| \| **1** \| **enriched** \| 40,6 \| 0,2 \| 40,7 \| 0,2 \| \| **2** \| **control** \| 40,5 \| 0,3 \| 40,8 \| 0,2 \| \| **2** \| **enriched** \| 40,5 \| 0,1 \| 40,7 \| 0,2 \| \| **3** \| **control** \| 40,8 \| 0,2 \| 40,9 \| 0,2 \| \| **3** \| **enriched** \| 40,7 \| 0,2 \| 40,9 \| 0,2 \| \| **4** \| **control** \| 40,7 \| 0,2 \| 40,9 \| 0,2 \| \| **4** \| **enriched** \| 40,7 \| 0,4 \| 41,0 \| 0,2 \| \| **5** \| **control** \| 41,2 \| 0,1 \| 41,2 \| 0,2 \| \| **5** \| **enriched** \| 41,0 \| 0,1 \| 41,2 \| 0,2 \| \| **6** \| **control** \| 41,0 \| 0,1 \| 41,2 \| 0,3 \| \| **6** \| **enriched** \| 41,0 \| 0,3 \| 41,1 \| 0,2 \| \| **7** \| **control** \| 40,9 \| 0,2 \| 41,0 \| 0,2 \| \| **7** \| **enriched** \| 40,8 \| 0,3 \| 41,0 \| 0,3 \| \| **14** \| **control** \| 41,0 \| 0,2 \| 41,1 \| 0,2 \| \| **14** \| **enriched** \| 41,1 \| 0,1 \| 41,1 \| 0,2 \| \| **21** \| **control** \| 41,0 \| 0,2 \| 41,0 \| 0,3 \| \| **21** \| **enriched** \| 40,9 \| 0,4 \| 41,1 \| 0,3 \| \| **28** \| **control** \| 40,9 \| 0,3 \| 41,0 \| 0,2 \| \| **28** \| **enriched** \| 41,0 \| 0,2 \| 41,0 \| 0,1 \| \| **35** \| **control** \| 40,9 \| 0,3 \| 41,2 \| 0,2 \| \| **35** \| **enriched** \| 41,2 \| 0,1 \| 41,2 \| 0,2 \| |
| --- | --- | --- | --- | --- | --- | --- | --- | --- | --- | --- | --- | --- | --- | --- | --- | --- | --- | --- | --- | --- | --- | --- | --- | --- | --- | --- | --- | --- | --- | --- | --- | --- | --- | --- | --- | --- | --- | --- | --- | --- | --- | --- | --- | --- | --- | --- | --- | --- | --- | --- | --- | --- | --- | --- | --- | --- | --- | --- | --- | --- | --- | --- | --- | --- | --- | --- | --- | --- | --- | --- | --- | --- | --- | --- | --- | --- | --- | --- | --- | --- | --- | --- | --- | --- | --- | --- | --- | --- | --- | --- | --- | --- | --- | --- | --- | --- | --- | --- | --- | --- | --- | --- | --- | --- | --- | --- | --- | --- | --- | --- | --- | --- | --- | --- | --- | --- | --- | --- | --- | --- | --- | --- | --- | --- | --- | --- | --- | --- | --- | --- | --- | --- | --- | --- | --- | --- | --- | --- | --- | --- | --- | --- | --- | --- | --- | --- | --- | --- | --- | --- | --- | --- | --- | --- | --- | --- | --- | --- | --- | --- | --- | --- |

| **Table S3** Mean group activity (± S.D.) throughout rearing period from day 1 to day 34 in enriched and control pens. (Environment: control – without elevated platforms, enriched – with elevated platform) from HH (hatchery-hatched) and OH (on-farm hatched) broiler chickens   \|  \|  \| **HH** \| \| **OH** \| \| \| --- \| --- \| --- \| --- \| --- \| --- \| \| **Age in days** \| **Environment** \| **Mean** \| **SD** \| **Mean** \| **SD** \| \| **1** \| **control** \| 143859 \| 9968 \| 161628 \| 7799 \| \| **1** \| **enriched** \| 233433 \| 23247 \| 229936 \| 34682 \| \| **2** \| **control** \| 150131 \| 13188 \| 171411 \| 10391 \| \| **2** \| **enriched** \| 238426 \| 25055 \| 234637 \| 34885 \| \| **3** \| **control** \| 156481 \| 16106 \| 182071 \| 12561 \| \| **3** \| **enriched** \| 246233 \| 25323 \| 243281 \| 35996 \| \| **4** \| **control** \| 171544 \| 17021 \| 195737 \| 14166 \| \| **4** \| **enriched** \| 253253 \| 22742 \| 252960 \| 36871 \| \| **5** \| **control** \| 170394 \| 15562 \| 197840 \| 16829 \| \| **5** \| **enriched** \| 261554 \| 24904 \| 257695 \| 37424 \| \| **6** \| **control** \| 180601 \| 15540 \| 206430 \| 13886 \| \| **6** \| **enriched** \| 268115 \| 25219 \| 265740 \| 35495 \| \| **7** \| **control** \| 195608 \| 18342 \| 221968 \| 16506 \| \| **7** \| **enriched** \| 281310 \| 29132 \| 276575 \| 35826 \| \| **8** \| **control** \| 199860 \| 18860 \| 223818 \| 15148 \| \| **8** \| **enriched** \| 284243 \| 30169 \| 278810 \| 32792 \| \| **9** \| **control** \| 207466 \| 20321 \| 232421 \| 13135 \| \| **9** \| **enriched** \| 288999 \| 29443 \| 284144 \| 34736 \| \| **10** \| **control** \| 219258 \| 20150 \| 245377 \| 16717 \| \| **10** \| **enriched** \| 298781 \| 28309 \| 294153 \| 38918 \| \| **11** \| **control** \| 225035 \| 15965 \| 253284 \| 15617 \| \| **11** \| **enriched** \| 310114 \| 26351 \| 299628 \| 35301 \| \| **12** \| **control** \| 234327 \| 16921 \| 260041 \| 15530 \| \| **12** \| **enriched** \| 316769 \| 27183 \| 306960 \| 31367 \| \| **13** \| **control** \| 243806 \| 16861 \| 266295 \| 15306 \| \| **13** \| **enriched** \| 320971 \| 26466 \| 309814 \| 34905 \| \| **14** \| **control** \| 255758 \| 16021 \| 278834 \| 14426 \| \| **14** \| **enriched** \| 330697 \| 27687 \| 320559 \| 34360 \| \| **15** \| **control** \| 264288 \| 16346 \| 285636 \| 16340 \| \| **15** \| **enriched** \| 332953 \| 29267 \| 325988 \| 32641 \| \| **16** \| **control** \| 268294 \| 25785 \| 295684 \| 16712 \| \| **16** \| **enriched** \| 338846 \| 29279 \| 331572 \| 29347 \| \| **17** \| **control** \| 278119 \| 20386 \| 303998 \| 17499 \| \| **17** \| **enriched** \| 344226 \| 28464 \| 336789 \| 28609 \| \| **18** \| **control** \| 289106 \| 18980 \| 310681 \| 17315 \| \| **18** \| **enriched** \| 350743 \| 26707 \| 343115 \| 32393 \| \| **19** \| **control** \| 295826 \| 17840 \| 316446 \| 17330 \| \| **19** \| **enriched** \| 353137 \| 27492 \| 345693 \| 26672 \| \| **20** \| **control** \| 300376 \| 19480 \| 323065 \| 16287 \| \| **20** \| **enriched** \| 358717 \| 28688 \| 351848 \| 27397 \| \| **21** \| **control** \| 303400 \| 17901 \| 327710 \| 18444 \| \| **21** \| **enriched** \| 360963 \| 29098 \| 351145 \| 27903 \| \| **22** \| **control** \| 301053 \| 16445 \| 324626 \| 15768 \| \| **22** \| **enriched** \| 360287 \| 25629 \| 350327 \| 26550 \| \| **23** \| **control** \| 307739 \| 17595 \| 315420 \| 37231 \| \| **23** \| **enriched** \| 363575 \| 25489 \| 354697 \| 25595 \| \| **24** \| **control** \| 311230 \| 18018 \| 319923 \| 38957 \| \| **24** \| **enriched** \| 365993 \| 25388 \| 355890 \| 27022 \| \| **25** \| **control** \| 307852 \| 15654 \| 331983 \| 15397 \| \| **25** \| **enriched** \| 367979 \| 26861 \| 360947 \| 22047 \| \| **26** \| **control** \| 308641 \| 17249 \| 319851 \| 42560 \| \| **26** \| **enriched** \| 367184 \| 25303 \| 360069 \| 23876 \| \| **27** \| **control** \| 302945 \| 21075 \| 317415 \| 38456 \| \| **27** \| **enriched** \| 367362 \| 21611 \| 360672 \| 23346 \| \| **28** \| **control** \| 310385 \| 19849 \| 321071 \| 39754 \| \| **28** \| **enriched** \| 370464 \| 21001 \| 364349 \| 21399 \| \| **29** \| **control** \| 299796 \| 16348 \| 310055 \| 39603 \| \| **29** \| **enriched** \| 364762 \| 20262 \| 359570 \| 24340 \| \| **30** \| **control** \| 306065 \| 15958 \| 315448 \| 39517 \| \| **30** \| **enriched** \| 364342 \| 20490 \| 361925 \| 22983 \| \| **31** \| **control** \| 309616 \| 17164 \| 319074 \| 37878 \| \| **31** \| **enriched** \| 366103 \| 18112 \| 362723 \| 24775 \| \| **32** \| **control** \| 304487 \| 17195 \| 327265 \| 14998 \| \| **32** \| **enriched** \| 364663 \| 15083 \| 359356 \| 22962 \| \| **33** \| **control** \| 307898 \| 15336 \| 330683 \| 14697 \| \| **33** \| **enriched** \| 363808 \| 16225 \| 363787 \| 24298 \| \| **34** \| **control** \| 318727 \| 20248 \| 333121 \| 22766 \| \| **34** \| **enriched** \| 372868 \| 19931 \| 370483 \| 23085 \| |
| --- | --- | --- | --- | --- | --- | --- | --- | --- | --- | --- | --- | --- | --- | --- | --- | --- | --- | --- | --- | --- | --- | --- | --- | --- | --- | --- | --- | --- | --- | --- | --- | --- | --- | --- | --- | --- | --- | --- | --- | --- | --- | --- | --- | --- | --- | --- | --- | --- | --- | --- | --- | --- | --- | --- | --- | --- | --- | --- | --- | --- | --- | --- | --- | --- | --- | --- | --- | --- | --- | --- | --- | --- | --- | --- | --- | --- | --- | --- | --- | --- | --- | --- | --- | --- | --- | --- | --- | --- | --- | --- | --- | --- | --- | --- | --- | --- | --- | --- | --- | --- | --- | --- | --- | --- | --- | --- | --- | --- | --- | --- | --- | --- | --- | --- | --- | --- | --- | --- | --- | --- | --- | --- | --- | --- | --- | --- | --- | --- | --- | --- | --- | --- | --- | --- | --- | --- | --- | --- | --- | --- | --- | --- | --- | --- | --- | --- | --- | --- | --- | --- | --- | --- | --- | --- | --- | --- | --- | --- | --- | --- | --- | --- | --- | --- | --- | --- | --- | --- | --- | --- | --- | --- | --- | --- | --- | --- | --- | --- | --- | --- | --- | --- | --- | --- | --- | --- | --- | --- | --- | --- | --- | --- | --- | --- | --- | --- | --- | --- | --- | --- | --- | --- | --- | --- | --- | --- | --- | --- | --- | --- | --- | --- | --- | --- | --- | --- | --- | --- | --- | --- | --- | --- | --- | --- | --- | --- | --- | --- | --- | --- | --- | --- | --- | --- | --- | --- | --- | --- | --- | --- | --- | --- | --- | --- | --- | --- | --- | --- | --- | --- | --- | --- | --- | --- | --- | --- | --- | --- | --- | --- | --- | --- | --- | --- | --- | --- | --- | --- | --- | --- | --- | --- | --- | --- | --- | --- | --- | --- | --- | --- | --- | --- | --- | --- | --- | --- | --- | --- | --- | --- | --- | --- | --- | --- | --- | --- | --- | --- | --- | --- | --- | --- | --- | --- | --- | --- | --- | --- | --- | --- | --- | --- | --- | --- | --- | --- | --- | --- | --- | --- | --- | --- | --- | --- | --- | --- | --- | --- | --- | --- | --- | --- | --- | --- | --- | --- | --- | --- | --- | --- | --- | --- | --- | --- | --- | --- | --- | --- | --- | --- | --- | --- | --- | --- | --- | --- | --- | --- | --- | --- | --- | --- | --- | --- | --- | --- | --- | --- | --- | --- | --- | --- | --- | --- | --- | --- | --- | --- | --- | --- | --- | --- | --- | --- | --- | --- | --- | --- | --- | --- | --- | --- | --- | --- | --- | --- | --- | --- | --- | --- | --- | --- | --- | --- | --- | --- | --- | --- | --- | --- | --- | --- | --- | --- | --- | --- | --- | --- | --- | --- |

| **Table S4** Mean relative usage (± S.D.) of platforms for OH chickens (on-farm) or HH chickens (hatchery-hatched) during light phase and the dark phase.   \|  \|  \| **HH** \| \| **OH** \| \| \| --- \| --- \| --- \| --- \| --- \| --- \| \| **Period** \| **Week of life** \| **Mean** \| **SD** \| **Mean** \| **SD** \| \| **dark** \| 1 \| 0,000 \| 0,000 \| 0,003 \| 0,010 \| \| **dark** \| 2 \| 0,026 \| 0,047 \| 0,044 \| 0,064 \| \| **dark** \| 3 \| 0,170 \| 0,084 \| 0,206 \| 0,087 \| \| **dark** \| 4 \| 0,266 \| 0,069 \| 0,306 \| 0,067 \| \| **dark** \| 5 \| 0,281 \| 0,064 \| 0,322 \| 0,057 \| \| **light** \| 1 \| 0,000 \| 0,002 \| 0,001 \| 0,007 \| \| **light** \| 2 \| 0,121 \| 0,086 \| 0,147 \| 0,099 \| \| **light** \| 3 \| 0,209 \| 0,084 \| 0,247 \| 0,090 \| \| **light** \| 4 \| 0,233 \| 0,082 \| 0,251 \| 0,078 \| \| **light** \| 5 \| 0,213 \| 0,069 \| 0,232 \| 0,070 \| |
| --- | --- | --- | --- | --- | --- | --- | --- | --- | --- | --- | --- | --- | --- | --- | --- | --- | --- | --- | --- | --- | --- | --- | --- | --- | --- | --- | --- | --- | --- | --- | --- | --- | --- | --- | --- | --- | --- | --- | --- | --- | --- | --- | --- | --- | --- | --- | --- | --- | --- | --- | --- | --- | --- | --- | --- | --- | --- | --- | --- | --- | --- | --- | --- | --- | --- | --- | --- | --- | --- | --- | --- | --- |

| **Table S5** Descriptive presentation of the feed conversion of the different treatment groups (Hatching system: HH – hatchery-hatched, OH – on-farm hatched; Environment: control – without elevated platforms, enriched – with elevated platform) | | | |
| --- | --- | --- | --- |
| **Hatching system *environment** | **Mean feed intake [kg/animal/trial]** | **Weight [kg]** | **FCR** |
| **HH*control** | 3.20 | 2.4 | 1.3 |
| **HH*enriched** | 3.23 | 2.4 | 1.3 |
| **OH*control** | 3.26 | 2.4 | 1.3 |
| **OH*enriched** | 3.23 | 2.4 | 1.3 |
